# Decoding the Rejection Code: Understanding Why Articles Get Axed

**DOI:** 10.7759/cureus.56920

**Published:** 2024-03-25

**Authors:** Vishal V Bhende, Tanishq S Sharma, Mathangi Krishnakumar, Anikode Subramanian Ramaswamy, Kanchan Bilgi, Saptak P Mankad

**Affiliations:** 1 Pediatric Cardiac Surgery, Bhanubhai and Madhuben Patel Cardiac Centre, Shree Krishna Hospital, Bhaikaka University, Karamsad, IND; 2 Anaesthesiology, St John’s Medical College Hospital, Bengaluru, IND; 3 Pathology, PES Institute of Medical Sciences and Research, Kuppam, IND; 4 Neuro-anaesthesiology, People Tree Hospitals, Bengaluru, IND; 5 Internal Medicine, Dev Medical Hospital, Vadodara, IND

**Keywords:** peer reviews, systematic review, publication success, manuscript rejection, medical writing

## Abstract

In the competitive arena of medical publishing, manuscript rejection remains a significant barrier to disseminating research findings. This editorial delves into the multifaceted nature of manuscript rejection, elucidating common reasons and proposing actionable strategies for authors to enhance their chances of acceptance. Key rejection factors include a mismatch with journal scope, lack of novelty, methodological flaws, inconclusive results, ethical issues, poor presentation, data inaccessibility, author misconduct, and plagiarism. Ethical lapses, such as lacking informed consent, or submissions fraught with grammatical errors, further doom manuscripts. In addressing these pitfalls, authors are advised to ensure content originality, methodological rigor, ethical compliance, and clear presentation. Aligning the manuscript with the journal’s audience, scope, and editorial standards is crucial, as is professional conduct and responsiveness to feedback. Leveraging technological tools for citation management, grammar checking, and plagiarism detection can also significantly bolster manuscript quality. Ultimately, understanding and addressing common rejection reasons can empower authors to improve their submissions, contributing to the advancement of medical knowledge and their professional growth.

## Editorial

**Figure 1:**
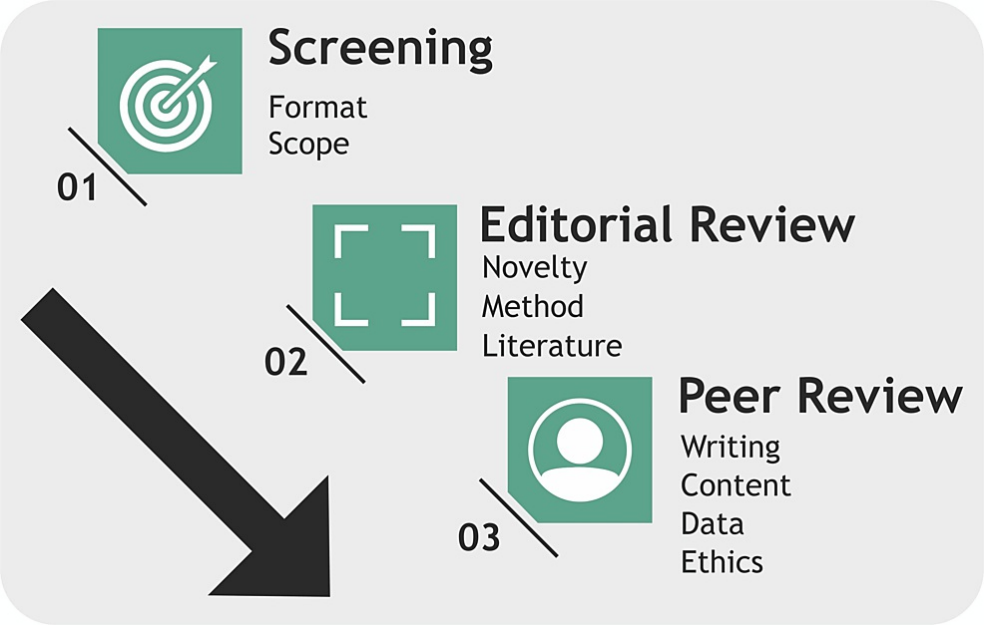
The process of decoding rejection. Image credits: Dr. Mathangi Krishnakumar.

Publishing in scholarly journals has become indispensable for career progression, achieving promotions, and securing research grants. Scholarly articles serve as a vital means for verifying research, sharing results, and participating in intellectual exchanges with peers. They also contribute to earning acclaim and visibility for both the researcher and their associated organization [1]. It is widely acknowledged that authors invest significant time, effort, and resources into conducting their studies, preparing their manuscripts for potential publication, and selecting an appropriate journal for their work. Nevertheless, the biggest hurdle in the process is manuscript rejection [2,3]. The whole experience can be nerve-wracking and every researcher or clinician has this obstacle to cross, with each experience giving an opportunity to learn and grow [4]. The digital era and automation have made article submissions easier and more accessible. However, more than 50% of the articles submitted to top medical journals get rejected even before undergoing a peer review process [5,6]. In this editorial, we will explore the multifaceted nature of rejection in medical manuscript submissions, dissecting its underlying causes and offering practical strategies for authors to navigate this challenging terrain. Figure 1 depicts decoding rejection.

Rejection letters from medical journals often contain generic explanations that cite reasons such as lack of novelty, failure to meet editorial standards, and mismatch with the journal’s scope and readership. While these explanations may seem discouraging at first glance, they provide valuable insights into the shortcomings of the manuscript and areas that require improvement. In the competitive landscape of medical literature, originality, accuracy, and clarity are paramount. Manuscripts that fail to offer novel insights, contain factual inaccuracies, or lack clear and coherent arguments are unlikely to garner acceptance. Authors must ensure that their submissions contribute unique perspectives, adhere to rigorous research methodologies, and present findings in a lucid and compelling manner. Additionally, the significance of the research and its implications for clinical practice should be clearly articulated to resonate with the journal’s readership [3,4]. The most common causes with examples are discussed below [7].

Mismatch with the journal’s scope

Example: A manuscript detailing the effects of yoga on mental health submitted to a journal that focuses on surgical innovations.

Journals typically seek manuscripts that fit within their specific area of interest to maintain coherence in their content and serve their target audience effectively. Before submission, authors should thoroughly review the journal’s aims and scope to ensure alignment [4,7].

Lack of novelty or originality

Example: A study presenting findings on the efficacy of aspirin in reducing cardiovascular risk.

Despite being methodologically sound, this manuscript can be rejected due to its lack of originality. The efficacy of aspirin in cardiovascular protection is a well-documented phenomenon, and the study fails to offer new insights or challenge existing knowledge, rendering it unsuitable for publication in a journal seeking innovative research. A study replicating established findings without providing new insights or advancements in the field may be rejected for lacking innovation. Journals seek contributions that push the boundaries of existing knowledge [8].

Poor methodological quality

Example: A clinical study on a new diabetes medication that lacked a control group.

The manuscript will be rejected because the absence of a control group compromised the study’s validity. Control groups are essential in clinical research to establish causality and ensure the reliability of the findings. Without this, the study’s results are questionable, making it unsuitable for publication. Sound research design is critical for acceptance [8].

Insignificant or inconclusive results

Example: Research investigating a potential link between a dietary supplement and improved cognitive function, which was performed on 10 subjects.

This manuscript can be rejected due to its inconclusive results. Research findings that do not provide clear, significant, or clinically relevant outcomes might be deemed insufficient for publication. Journals favor studies that offer definitive conclusions or meaningful advancements [8].

Ethical concerns

Example: A study involving human subjects that did not document informed consent.

The manuscript is likely to face rejection on ethical grounds, as it fails to provide evidence of informed consent from the participants. Ethical compliance, including informed consent, is a cornerstone of research involving human subjects, and its absence is a critical violation that journals take seriously. Adherence to ethical standards is non-negotiable [7].

Poor presentation and writing quality

Example: A manuscript riddled with grammatical errors and lacking a clear structure.

Despite potentially valuable findings, manuscripts can get rejected due to poor writing and presentation. The prevalence of grammatical errors and a lack of coherent structure impede the clarity of the research, making it difficult for reviewers and readers to engage with the content effectively. A clear and coherent presentation of research is essential for both review and publication [7,8].

Data accessibility and reproducibility issues

Example: A manuscript that did not include the raw data supporting its conclusions or provide detailed methodological procedures.

The manuscript can be rejected because it does not adhere to the principles of transparency and reproducibility. The absence of raw data and insufficient methodological detail prevent other researchers from verifying or replicating the study’s findings, which is fundamental for the advancement of scientific knowledge [7,8].

Author misconduct

Example: A manuscript that does not address the queries raised by the reviewer.

Professionalism in communication and adherence to submission protocols are non-negotiable in the realm of medical publishing. Authors should maintain courteous and respectful interactions with journal editors, adhere to submission deadlines, and respond promptly to editorial queries. Furthermore, receptiveness to feedback and a willingness to revise and improve upon initial submissions are indicative of a commitment to excellence in scholarly communication. By demonstrating professionalism and collaboration throughout the submission process, authors can enhance their chances of success [4].

Plagiarism

Example: A manuscript having content verbatim from a previous publication, not quoting relevant literature.

**Figure 2:**
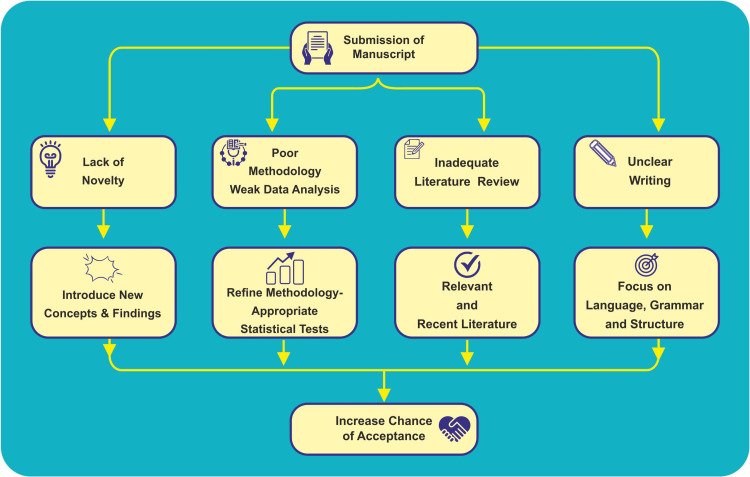
Strategies to avoid rejections. Image credits: Dr. Mathangi Krishnakumar.

Journals employ plagiarism detection tools to identify high levels of similarity. Manuscripts with significant overlap, including self-plagiarism, will be flagged. It is important to ensure originality and proper citation to avoid plagiarism issues [8]. Figure 2 depicts strategies to avoid rejection and crafting compelling manuscripts.

In light of the aforementioned considerations, authors can adopt several strategies to enhance the acceptability of their manuscripts in medical journals.

Content excellence

The manuscript should be unique in well-established subjects, bringing to light innovative perspectives and in-depth analyses. It is crucial to uphold the accuracy of your data and the reliability of your sources, as the strength of your manuscript relies on solid, well-researched foundations. Embed your unique voice within your writing, aiming for clarity and simplicity to ensure your message is conveyed without the need for overly complex jargon. Your manuscript should be well-organized, with a clear, logical flow and compelling narrative that naturally develops your arguments and keeps readers engaged. Additionally, meticulous proofreading and strict adherence to the formatting guidelines of your intended journal are essential, as even the smallest mistakes can undermine the credibility of your work [9-11].

Aligning with the scope

Ensure your manuscript aligns with the interests of the journal’s readers; a mismatch in content and audience preferences could affect its acceptance. Understand and embrace the journal’s specific style and themes, so your work fits well with what is already published. When interacting with the journal’s team, always be respectful and professional, as your conduct can greatly impact the review process. Pay close attention to the journal’s submission guidelines; ignoring them might be seen as disregarding the publication’s standards. Treat any feedback you receive as an opportunity to improve your manuscript; being open to making changes can significantly enhance its quality [12].

Elevating your manuscript

Enhance your expertise in your specialized field to create content that is both insightful and authoritative, making it stand out. Explore under-investigated niches within your discipline to challenge existing beliefs and introduce fresh viewpoints that captivate and stimulate your audience [9-11]. Organize your manuscript with great care, ensuring it leads readers seamlessly through your arguments with clear transitions and a coherent structure [11]. Thoroughly check all your claims and data, relying on credible sources to strengthen your case. Employ proofreading strategies and tools to eliminate mistakes, thereby improving your manuscript’s readability and professionalism [12]. View feedback and revisions as chances to grow, letting your work develop and improve with each round of critique [13]. Recognize that rejection is a natural part of the publishing process, and use each experience to refine your methods and sharpen your writing abilities for future endeavors.

Leveraging technological advancements

Technology offers a suite of tools to enhance your scientific writing process. From reference management software such as Zotero and Mendeley, which streamline citation organization, to grammar and style checkers such as Grammarly that polish your prose, these tools can significantly improve the quality of your manuscript. Plagiarism detectors ensure the originality of your work, while collaborative platforms facilitate efficient teamwork. Embracing these technological aids can free up more time for the core of your research, allowing you to focus on making impactful discoveries and sharing them with the academic community [12].

Conclusions

While rejection in medical manuscript submissions can be disheartening, it is not indicative of the inherent quality or merit of the work. By understanding the reasons for rejection and actively addressing areas of weakness, authors can improve the likelihood of acceptance in subsequent submissions. By adhering to rigorous research standards, aligning with journal requirements, and maintaining professionalism in authorial conduct, authors can navigate the peer review process with confidence and ultimately contribute to the advancement of medical knowledge. The pursuit of publication excellence in medical journals demands dedication, perseverance, and a commitment to continuous improvement.
